# Convalescent Plasma in a Patient with Protracted COVID-19 and Secondary Hypogammaglobulinemia Due to Chronic Lymphocytic Leukemia: Buying Time to Develop Immunity?

**DOI:** 10.3390/idr13040077

**Published:** 2021-09-27

**Authors:** Jaap L. J. Hanssen, Johan Stienstra, Stefan A. Boers, Cilia R. Pothast, Hans L. Zaaijer, Jennifer M. Tjon, Mirjam H. M. Heemskerk, Mariet C. W. Feltkamp, Sandra M. Arend

**Affiliations:** 1 Department of Infectious Diseases, Leiden University Medical Center, C5-P, Albinusdreef 2, 2333 ZA Leiden, The Netherlands; smarend@lumc.nl; 2 Department of Internal Medicine, Leiden University Medical Center, Albinusdreef 2, 2333 ZA Leiden, The Netherlands; j.stienstra@lumc.nl; 3 Department of Medical Microbiology, Leiden University Medical Center, E4-P, Albinusdreef 2, 2333 ZA Leiden, The Netherlands; sboer@lumc.nl (S.A.B.); MCVFeltkamp@lumc.nl (M.C.W.F.); 4 Department of Hematology, Leiden University Medical Center, C2-R, Albinusdreef 2, 2333 ZA Leiden, The Netherlands; CApothast@lumc.nl (C.R.P.); Jtjon@lumc.nl (J.M.T.); MHM@lumc.nl (M.H.M.H.); 5 Sanquin Blood Supply Foundation, Plesmanlaan 125, 1066 CX Amsterdam, The Netherlands; Hzaaijer@sanquin.nl

**Keywords:** chronic lymphocytic leukemia, convalescent plasma, COVID-19, in vitro T cell assay

## Abstract

It is not exactly clear yet which type of immune response prevails to accomplish viral clearance in coronavirus disease 2019 (COVID-19). Studying a patient with chronic lymphocytic leukemia and hypogammaglobulinemia who suffered from COVID-19 provided insight in the immunological responses after treatment with COVID-19 convalescent plasma (CCP). Treatment consisted of oxygen, repeated glucocorticosteroids and multiple dosages of CCP guided by antibody levels. Retrospectively performed humoral and cellular immunity analysis made clear that not every serological test for COVID-19 is appropriate for follow-up of sufficient neutralizing antibodies after CCP. In retrospect, we think that CCP merely bought time for this patient to develop an adequate cellular immune response which led to viral clearance and ultimately clinical recovery.

## 1. Introduction

Coronavirus disease 2019 (COVID-19), the illness caused by severe acute respiratory syndrome coronavirus 2 (SARS-CoV-2) primarily affects the respiratory system and is spread from person to person through respiratory particles. The immune system represents a double-edged sword in the pathogenesis of COVID-19, leading to viral clearance as well as deleterious hyperinflammation in severe cases. It is not clear yet which type of immune response prevails to accomplish viral clearance. In that regard, the clinical course of COVID-19 in patients with a specific immune disorder may shed light on the role of the different components of the immune system in this puzzling disease. Chronic lymphocytic leukemia (CLL) is a clonal B cell lymphoproliferative disorder characterized by the accumulation of mature lymphocytes in the peripheral blood, bone marrow, spleen and other lymphoid tissues. It is often associated with secondary hypogammaglobulinemia which is strongly correlated with the risk of infection and is often treated with immune globulins to support humoral immune responses. Additional immune defects may also occur, such as impaired function of natural killer cells and T cell exhaustion [1]. Two studies in patients with CLL who were hospitalized for COVID-19 reported a high case-fatality rate of 36% [2,3]. In general, the added value of COVID-19 convalescent plasma (CCP), containing excess of (neutralizing) severe acute respiratory syndrome coronavirus 2 (SARS-CoV-2) antibodies obtained from patients who recovered from COVID-19, for treating patients infected with SARS-CoV-2 has not yet crystallized, with inconsistent results between studies [4,5]. However, the situation may be different in CLL patients who cannot mount an efficient antibody response to novel antigens and might substantially benefit from CCP [6,7].

We present a patient with B-CLL and secondary hypogammaglobulinemia who suffered from protracted COVID-19. Based on failure to develop SARS-CoV-2 antibodies, he received CCP. Thereafter, rapid decline of SARS-CoV-2 antibody titer in association with clinical deterioration prompted repeated dosing of CCP. Only after the third dose of CCP SARS-CoV-2 the patient recovered followed by viral clearance, suggesting causality. However, additional evaluation of nucleocapsid (NC) protein- and spike (S) antigen-directed antibody responses as well as T cell responses against various SARS-CoV-2 antigens suggested a different model to explain the delayed cure, which may help to understand the value of CCP in immunocompromised patients.

## 2. Case Report

A 77-year-old man was diagnosed in 2016 with B-CLL RAI stage IV, Binet stage C. Six cycles of fludarabine and cyclophosphamide with rituximab resulted in 16 months of stable disease. In 2018 ibrutinib q.d. 420 mg was started for progressive disease, consisting of increased lymphocyte count up to 138 × 10^9^/L, lymphadenopathy and thrombocytopenia together with weight loss and night sweats. Ibrutinib induced cessation of progression, normalization of peripheral lymphocyte count and led to stable disease. In that year however, he suffered from recurrent respiratory tract infections and pneumonias due to secondary hypogammaglobulinemia which was diagnosed three months after the start of ibrutinib. Monthly subcutaneous immuneglobulin therapy and daily co-trimoxazole prophylaxis were started and was followed by normalization of serum IgG levels and reduced frequency of respiratory tract infections albeit with remaining pulmonary abnormalities on imaging. In September 2020 there was still no progression of B-CLL with lymphocytes of 3.3 × 10^9^/L, hemoglobine of 9.1 mmol/L and thrombocytes of 125 × 10^9^/L.

**Figure 1:**
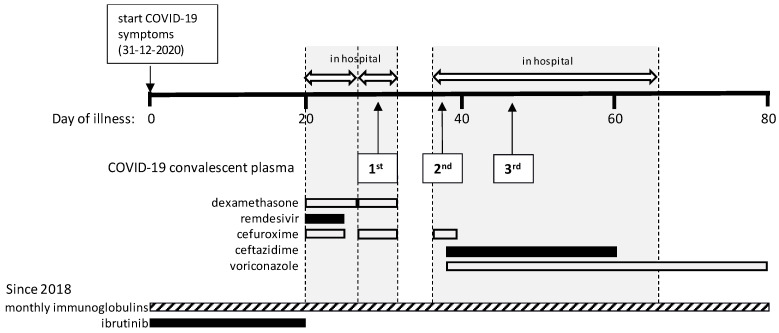
Timeline of a patient with CLL and prolonged COVID-19.

**Figure 2:**
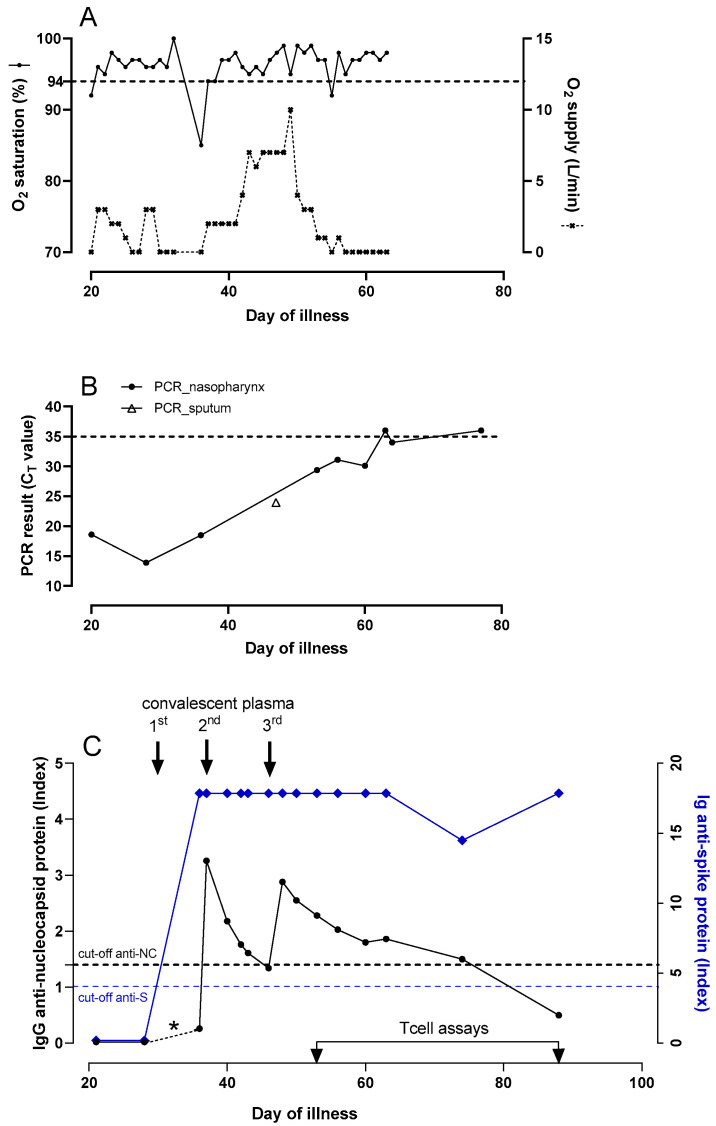
Clinical status, PCR results and serological responses in a patient with prolonged COVID-19. Time course of clinical status, PCR results and serological values. (**A**) Oxygen supplementation and saturation level during the admission periods. (**B**) PCR results of nasopharyngeal swabs and one sputum sample (Ct value means cycle threshold: a high value indicates a low amount or viral RNA and vice versa). The cut-off is indicated with an interrupted line. (**C**) Results of serological assays. Anti-NC was measured repeatedly during admission. The anti-S antibodies were measured only retrospectively. The cut-off values for both assays and the three time points of administration of convalescent plasma are indicated. The results of the T cell assays are shown in Figure 3 and Supplementary Figures S3 and S4.

On 31 December 2020, the patient developed fever and two days later reverse-transcription polymerase chain reaction (RT-PCR) testing on a nasopharyngeal swab was positive for SARS-CoV-2. During the ensuing weeks he remained febrile and had to be admitted on day 20 with progressive dyspnea and hypoxemia (Figure 1 and Figure 2A). The PCR tested positive again, with a cycle threshold (C_T_) value of 18.6 (Figure 2B). Laboratory results showed lymphocytes of 1.6 × 10^9^/L, hemoglobine of 8.4 mmol/L and thrombocytes of 165 × 10^9^/L, lymphocyte subsets were in the normal range, C-reactive protein (CRP) was 84 mg/L (normal value < 5 mg/L) and D-dimer was elevated. A chest CT showed extensive bilateral areas of ground-glass opacities and peri-bronchial consolidations, comprising about 60% of the total lung tissue, without signs of pulmonary embolism. There were no detectable levels of serum SARS-CoV-2 IgG antibodies to the NC protein (Alinity, Abbott Laboratories Diagnostics Division Abbott Park, IL 60064 USA), Figure 2C. Based on this result, ibrutinib was paused. According to the standard of care at that time, dexamethasone q.d. 6 mg during 5 days and remdesivir q.d. 100 mg during 1 week were administered besides oxygen. In addition, cefuroxime t.i.d. 750 mg was given empirically during five days because of possible bacterial pneumonia.

After seven days in the hospital the patient was discharged in stable condition, CRP was 18 mg/L, but he had to be readmitted one day later on day 28 after disease onset with recurrent hypoxemia, fever and chest pain. CRP was 44 mg/L and the chest CT showed no improvement and again no embolism. Treatment with dexamethasone q.d. 6 mg during 5 days and empirical cefuroxime t.i.d. 750 mg were started again. The PCR results remained strongly positive (C_T_ 13.9; Figure 2B) and serum anti-NC SARS-CoV-2 IgG was still undetectable. On day 29, 300 cc of COVID-19 convalescent plasma (CCP, containing at least 60 Alliance Units/mL of SARS-CoV-2 antibodies) was given with the aim to support viral clearance, after which the patient improved and could be discharged four days later.

A third admission followed four days later on day 36 with persisting episodes of fever, dyspnea and coughing. The SARS-CoV-2 PCR still tested positive (C_T_ 18.5), CRP was 118 mg/L and another chest CT showed increase of bilateral multifocal ground-glass consolidations and a small cavitating consolidation in the left upper lobe, but no signs of embolism. Again, cefuroxime t.i.d. 750 mg was given empirically for possible bacterial pneumonia. Despite administration of CCP eight days earlier, serum anti-NC SARS-CoV-2 IgG was negative (unfortunately no serum sample was available to demonstrate a positive titer in the interval), while anti-spike (S) antibody testing was not yet performed, on which the patient received a second dose of CCP. Repeated cultures of sputum showed *Aspergillus fumigatus* and *Pseudomonas aeruginosa* for which voriconazole, first day b.i.d. 400 mg followed by b.i.d 200 mg. and ceftazidime t.i.d. 1000 mg were started. A serum galactomannan test performed during this admission was negative. After this second dose of CCP, serum SARS-CoV-2 IgG anti-NC levels were monitored frequently (Figure 2C). On day 46, serum SARS-CoV-2 IgG anti-NC tested negative again, while the SARS-CoV-2 PCR still tested positive (C_T_ 24 in a sputum sample). At this moment the patient required 10 L of oxygen per minute to keep oxygen saturation above 90%, after which a third dose of CCP was administered. Thereafter, the anti-NC antibody titer decreased but remained in the positive range, the patient showed gradual improvement, oxygen therapy could be tapered and CRP levels started to decline. On day 63, the patient was discharged to a rehabilitation clinic where he gradually improved further and was sent home on day 79 with a negative PCR test result. His general condition kept improving during outpatient follow-up. The lymphocyte count gradually increased from day 60 and ibrutinib was restarted on day 83 when lymphocytes were 12 × 10^9^/L.

## 3. Additional Analyses

In retrospect, i.e., after the patient had recovered and was finally discharged from the hospital, the serological analyses were extended with more sensitive and more relevant spike (S) antibody measurements (SARS-CoV-2 total antibody ELISA WS-1396, Wantai Biologicals), and BK polyomavirus (BKPyV) antibody measurements to learn about background humoral antiviral immunity in this patient [8]. Prior to CCP administration no SARS-CoV-2 anti-S antibody activity was detected (Figure 2C), while BKPyV antibodies were present (Supplementary Figure S1). Since the first CCP administration, anti-S antibodies were detectable, while anti-NC antibodies were detectable after the second dose, to disappear again. After the third CCP dose anti-NC antibodies declined slowly to negative on day 88, while anti-S antibodies showed a late modest decline, followed by a rise suggesting autologous antibody production (Figure 2C). For comparison, the strong anti-S seroresponses were titrated, but did not show a sawtooth pattern in response to CCP administrations as was observed for the anti-NC antibodies (Supplementary Figure S2).

**Figure 3:**
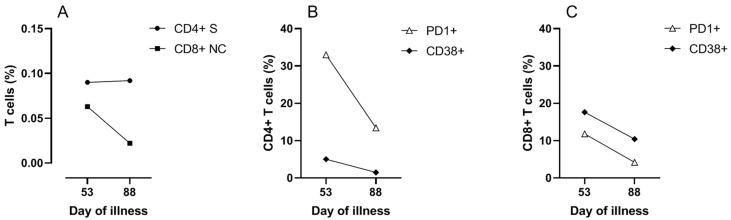
Reactivity and activation of SARS-CoV-2 specific T cells over time. Reactivity and activation of SARS-CoV-2 specific CD4 T cells over time. FACS analysis of T cells after overnight stimulation of PBMCs sampled on days 53 and 88 using peptides derived from SARS-CoV-2 spike (S) or nucleocapsid (NC) protein. (**A**) Reactivity of CD4 T cells towards the S protein (CD4 S) and reactivity of CD8 T cells towards the NC protein (CD8 NC) is shown as % CD154+TNFα+ of total CD4+ T cells, or as % CD137+IFNγ+ of total CD8+ T cells, respectively. (**B**) Frequency of activation marker (PD1 and CD38) positive SARS-CoV-2 reactive CD4+ T cells over time. (**C**) Frequency of activation marker (PD1 and CD38) positive SARS-CoV-2 reactive CD8+ T cells over time.

To investigate the contribution of cellular immune responses to the clinical course and viral clearance, SARS-CoV-2 specific CD4 and CD8 T cell responses were measured on day 53. Peripheral blood mononuclear cells were stimulated overnight with overlapping 15-mer peptide pools of the structural proteins of membrane (M), nucleocapsid (NC) and spike (S) proteins (Miltenyi Biotec b.v., Leiden, The Netherlands). Flow cytometric analyses (FACS) were performed to determine the frequency and activation status of the T cells. More detailed methods are provided in the Supplementary Materials. The results demonstrated SARS-CoV-2 reactive CD4 T cells specific for M, NC and S protein, and SARS-CoV-2 reactive CD8 T cells predominantly specific for NC protein (Figure 3 and Figure S3). SARS-CoV-2 reactive T cells expressed the activation markers PD1 and CD38 indicating in vivo activation (Figure 3B,C). Cellular assays were repeated on day 88, four weeks after the last discharge and 25 days after the last positive PCR, which showed that SARS-CoV-2 specific T cells were still present (Figure 3A). The SARS-CoV-2 specific T cells and total T cells at day 88 were less activated compared to day 53, in agreement with decreased responses after clearance of the virus (Figure 3B,C and Figure S4).

SARS-CoV-2 molecular analysis was extended with whole genome sequencing (WGS) of all samples with sufficient viral load (C_T_ < 30), to monitor occurrence of mutations as the result of immunological pressure imposed by administration of CCP in this immunocompromised patient (Table S1). The initial SARS-CoV-2 genome sequence belonged to the B.1.1.209 lineage [9], which was prevalent in The Netherlands at the time of infection in December 2020, with a total of 24 nucleotide substitutions throughout the genome compared to the original Wuhan-1 strain (10 synonymous and 14 non-synonymous; Table S1). During follow-up, non-synonymous substitutions detected in at least two consecutive samples were observed at positions 5178 and 5184, resulting in T820I and P822L amino acid-replacements located in NSP3 involved in viral replication, but no non-synonymous mutations in the S protein.

## 4. Discussion

This patient with hypogammaglobulinemia secondary to B-CLL had a prolonged course of COVID-19 associated with ongoing viral replication. Persistent clinical improvement and eventually viral clearance occurred after three admissions and three administrations of CCP. While it cannot be excluded that he would have recovered without any CCP treatment, this seems unlikely given the late progressive respiratory failure with persistent high viral load while the coinfections with *Aspergillus* and *Pseudomonas* were adequately treated. First, the discontinuation of ibrutinib and treatment with dexamethasone in our patient are discussed, followed by an evaluation of the humoral and cellular immune responses and the place of vaccination in this setting.

Early during the first admission, 21 days after onset of disease, ibrutinib was discontinued because at that time there was no detectable SARS-CoV-2 antibody response. This was done under the assumption that the inhibition by ibrutinib of Bruton tyrosine kinase in B cells and of interleukin-2-inducible T cell kinase in T cells impairs B and T cell responses, which are both needed for viral clearance. In retrospect, however, it can be questioned whether discontinuation of ibrutinib was necessary or may even have been disadvantageous. In patients with CLL, treatment with ibrutinib has been associated with partial reconstitution of humoral and cellular immune functions and fewer infections [10,11], although the risk of Aspergillosis is initially increased [12]. Interestingly, positive effects of ibrutinib on the course of COVID-19 have been reported in patients with or without hematological disease [3,13,14]. At the time of this writing, several randomized trials of ibrutinib as therapeutic modality are even under way, aimed to lessen the hyperimmune response of severe COVID-19 in immunocompetent patients with a high level of inflammation [9]. In patients with CLL, experts in the field caution against abrupt discontinuation of ibrutinib [15].

Our patient received dexamethasone during the first two admissions, which was standard of care in our hospital for severe COVID-19 at the time, based on reports of a positive effect on survival of severe cases through dampening of an inappropriate hyperinflammatory response [16,17]. However, glucocorticosteroids are known to inhibit various innate and adaptive immune responses and induce apoptosis of lymphocytes which can impair the development of an adaptive immune response and lead to prolonged viral shedding. The latter is a known undesired effect of treatment with dexamethasone. In patients such as described here with relatively low inflammatory parameters in association with high viral load late in the disease, the contribution of inflammation to the pathogenesis may have been less than that of virus-induced damage. In retrospect, the negative effects of dexamethasone probably outweighed the potential beneficial effect. In patients with an already impaired immune response the risk of impaired viral clearance as a result of dexamethasone should be carefully weighed against the possible advantageous anti-inflammatory effects.

Both humoral and cellular responses contribute to clearance of infection with SARS-CoV-2, although there is a large interindividual variation in the kinetics of responses [18]. Regarding humoral immunity, our patient was still seronegative three weeks into illness, prompting treatment with CCP. Interestingly, the index of reported neutralizing antibodies declined rapidly after the first two doses of CCP, which prompted to give additional doses of CCP. Only in retrospect it was found that the reported antibody index represented anti-NC antibodies while the clinically relevant anti-S protein antibody index remained high already after the first dose of CCP. This could be due to lower anti-NC than anti-S IgG concentrations in CCP, more rapid clearance of anti-NC antibodies through complexing with excess of NC protein, or a lower sensitivity of the anti-NC antibody assay. The remarkable pattern difference between anti-NC (sawtooth) and anti-S (flat) antibody responses observed here suggests differences in antibody kinetics against these structural antigens within this patient or it could reflect a difference in assay sensitivity. The clinical importance of this observation is not clear since it is the anti-S response that is responsible for neutralizing and clearance of virus.

At the time of clinical improvement and progressively declining viral load, broad SARS-CoV-2-specific CD4+ and CD8+ T cell responses against various antigens were detectable, reflecting that the patient’s own immune system was capable of mounting such responses. Taken together, these findings suggest that CCP in combination with oxygen therapy merely bought time until autologous T and possibly B cell responses developed, which are indispensable for viral clearance and cure. It is not yet known whether T helper cells, cytotoxic T cells and antibodies are all essential for cure or that there is a redundancy. In a macaque infection model, depletion of CD8+ T cells in convalescent macaques abrogated natural immunity against re-challenge, suggesting an important role of CD8+ T cells for long term protection [19]. Given that antibodies cannot reach an intracellular virus, it is plausible that CD8+ T cells are vital for viral clearance. While the T cell assays described above may not be generally available, more robust less laborious assays have been developed for detection of T cell responses to SARS-CoV-2 antigens [20].

Several reviews and meta-analyses showed that there is no clear benefit of CCP in immunocompetent patients who are admitted to the hospital with COVID-19 [4,5]. However, early administration of CCP within seven days from symptoms onset may generate the greatest chance of success [21,22]. In addition, several case series suggest it can be beneficial in patients with B-CLL and humoral immune dysfunction [21]. That CCP also leads to better outcomes in immunocompetent patients only when given very early in disease development [4,5] is probably explained by the fact that when administered in a later stage of the disease ‘autologous’ seroconversion already took place. Thus, the value of CCP in COVID-19, if any, depends heavily on timing. Interestingly, patients with a more severe SARS-CoV-2 infection even had a stronger antibody response to the spike and nucleocapsid protein [23]. This counterintuitive finding was explained by SARS-CoV-2-directed antibodies that functionally block the production of the mild disease-associated interferon-stimulated gene-expressing cells [24].

The mechanism of action that underlies the effect of CCP is thought to be the binding of neutralizing antibodies to the receptor binding domain of the viral spike antigen, thus inhibiting virus entry into target cells [21]. CCP is manufactured and calibrated based on the level of in vitro neutralizing antibodies. In the patient described here, the measured anti-NC titers seemed to correlate better with the clinical course than the anti-S titer and maybe reflected the disease activity more than that it represented a sufficient neutralizing antibody response. This case illustrates that awareness of the difference between anti-S and anti-NC responses can be clinically relevant as we found out only retrospectively and anti-NC titers are inappropriate for guiding therapy with CCP. In our hospital, serology is now offered separately for post-vaccination status (anti-S) and post-infection (anti-NC).

When clinical improvement upon the first CCP-administration was not observed, there were concerns that repeated CCP treatment might result in selection of a mutated virus escaping from neutralization by convalescent antibodies. However, WGS analysis revealed that no nucleotide mutations in the S gene occurred during CCP treatment, and only four nonsynonymous substitutions were detected in the other viral genome regions during follow-up, especially in NSP3 that is not included in the virus particle but involved in the intracellular virus replication complex.

Patients with various immune disorders now ask whether they can and should be vaccinated against COVID-19. For most patients the answer is yes, although the quality and quantity of the immune response may be suboptimal and may also depend on the specific vaccine. For example, cancer patients mounted a lower response to vaccination against COVID-19 and patients on ibrutinib responded poorly to influenza vaccination [25,26]. Patients with CLL, especially those on ibrutinib show poor responses to mRNA COVID-19 vaccination [27,28]. However, it may be wiser to postpone vaccination in situations of severe immune suppression such as early after transplantation or after use of B cell depleting therapies which virtually shut down humoral responses. In patients with CLL, CD4+ T cells show markedly impaired helper activity, which is the main cause of the frequent hypogammaglobulinemia and contributes to the poor response to vaccines, but any response may be better than none at all [1]. Ongoing studies measuring post-vaccination anti-S antibody and T cell responses and clinical follow up data in various populations will shed more light on what level of protection can be attained in various immunocompromised populations.

CLL patients with hypogammaglobulinemia are often treated with immunoglobulins. At present, commercial immunoglobulin products do not yet contain adequate levels of protective antibodies to prevent COVID-19 but this may change when more donors become seropositive due to vaccination and natural infection. An intravenous immunoglobulin product from donors who recovered from COVID-19 (named COVIg) is now available in The Netherlands.

Based on the association between the clinical course and humoral and cellular responses, we now think that CCP together with oxygen therapy merely bought time till the late development of virus-specific T cell responses, which ultimately led to viral clearance and cure. The contribution of the additional doses of CCP remains questionable. We did not find development of escape variants during CCP treatment. Further studies are needed to correlate viral and inflammatory parameters, as well as humoral and cellular immune responses to the clinical course, in order to provide a stronger foundation for truly individualized therapy for COVID-19 patients. 

## Supplementary Materials

The following are available online at https://www.mdpi.com/article/10.3390/idr13040077/s1, Figure S1. Serology for BK polyomavirus and seasonal coronavirus 229E-N. Figure S2. Anti-S antibodies (Wantai) in dilutions of plasma. Figure S3. FACS analysis of T cells obtained after overnight stimulation of PBMCs. Figure S4. Activation marker expression on CD4+ and CD8+ T cells. Table S1. Whole genome sequencing of all nasopharyngeal samples with sufficient viral load (C_T_ < 30).

## References

[1] Forconi F., Moss P. (2015). Perturbation of the normal immune system in patients with CLL. Blood.

[2] Mato A.R., Roeker L.E., Lamanna N., Allan J.N., Leslie L., Pagel J.M., Patel K., Osterborg A., Wojenski D., Kamdar M. (2020). Outcomes of COVID-19 in patients with CLL: A multicenter international experience. Blood.

[3] Scarfo L., Chatzikonstantinou T., Rigolin G.M., Quaresmini G., Motta M., Vitale C., Garcia-Marco J.A., Hernandez-Rivas J.A., Miras F., Baile M. (2020). COVID-19 severity and mortality in patients with chronic lymphocytic leukemia: A joint study by ERIC, the European Research Initiative on CLL, and CLL Campus. Leukemia.

[4] Janiaud P., Axfors C., Schmitt A.M., Gloy V., Ebrahimi F., Hepprich M., Smith E.R., Haber N.A., Khanna N., Moher D. (2021). Association of Convalescent Plasma Treatment With Clinical Outcomes in Patients With COVID-19: A Systematic Review and Meta-analysis. JAMA.

[5] Vegivinti C.T.R., Pederson J.M., Saravu K., Gupta N., Evanson K.W., Kamrowski S., Schmidt M., Barrett A., Trent H., Dibas M. (2021). Efficacy of convalescent plasma therapy for COVID-19: A systematic review and meta-analysis. J. Clin. Apher..

[6] Niu A., McDougal A., Ning B., Safa F., Luk A., Mushatt D.M., Nachabe A., Zwezdaryk K.J., Robinson J., Peterson T. (2020). COVID-19 in allogeneic stem cell transplant: High false-negative probability and role of CRISPR and convalescent plasma. Bone Marrow Transplant..

[7] Ormazabal Velez I., Indurain Bermejo J., Espinoza Perez J., Imaz Aguayo L., Delgado Ruiz M., Garcia-Erce J.A. (2021). Two patients with rituximab associated low gammaglobulin levels and relapsed covid-19 infections treated with convalescent plasma. Transfus. Apher. Sci..

[8] Kamminga S., van der Meijden E., Feltkamp M.C.W., Zaaijer H.L. (2018). Seroprevalence of fourteen human polyomaviruses determined in blood donors. PLoS ONE.

[9] Study of Oral Ibrutinib Capsules to Assess Respiratory Failure in Adult Participants With Severe Acute Respiratory Syndrome Coronavirus-2 (SARS-CoV-2) and Pulmonary Injury. https://clinicaltrials.gov/ct2/results?recrs=&cond=Covid19&term=ibrutinib&cntry=&state=&city=&dist=.

[10] Sun C., Tian X., Lee Y.S., Gunti S., Lipsky A., Herman S.E., Salem D., Stetler-Stevenson M., Yuan C., Kardava L. (2015). Partial reconstitution of humoral immunity and fewer infections in patients with chronic lymphocytic leukemia treated with ibrutinib. Blood.

[11] Yin Q., Sivina M., Robins H., Yusko E., Vignali M., O’Brien S., Keating M.J., Ferrajoli A., Estrov Z., Jain N. (2017). Ibrutinib Therapy Increases T Cell Repertoire Diversity in Patients with Chronic Lymphocytic Leukemia. J. Immunol..

[12] Blez D., Blaize M., Soussain C., Boissonnas A., Meghraoui-Kheddar A., Menezes N., Portalier A., Combadiere C., Leblond V., Ghez D. (2020). Ibrutinib induces multiple functional defects in the neutrophil response against Aspergillus fumigatus. Haematologica.

[13] Maynard S., Ros-Soto J., Chaidos A., Innes A., Paleja K., Mirvis E., Buti N., Sharp H., Palanicawandar R., Milojkovic D. (2021). The role of ibrutinib in COVID-19 hyperinflammation: A case report. Int. J. Infect. Dis..

[14] Thibaud S., Tremblay D., Bhalla S., Zimmerman B., Sigel K., Gabrilove J. (2020). Protective role of Bruton tyrosine kinase inhibitors in patients with chronic lymphocytic leukaemia and COVID-19. Br. J. Haematol..

[15] COVID-19 and CLL: Frequently Asked Questions. https://hematology.org/covid-19/covid-19-and-cll.

[16] Group R.C., Horby P., Lim W.S., Emberson J.R., Mafham M., Bell J.L., Linsell L., Staplin N., Brightling C., Ustianowski A. (2021). Dexamethasone in Hospitalized Patients with Covid-19. N. Engl. J. Med..

[17] Tomazini B.M., Maia I.S., Cavalcanti A.B., Berwanger O., Rosa R.G., Veiga V.C., Avezum A., Lopes R.D., Bueno F.R., Silva M. (2020). Effect of Dexamethasone on Days Alive and Ventilator-Free in Patients With Moderate or Severe Acute Respiratory Distress Syndrome and COVID-19: The CoDEX Randomized Clinical Trial. JAMA.

[18] Dan J.M., Mateus J., Kato Y., Hastie K.M., Yu E.D., Faliti C.E., Grifoni A., Ramirez S.I., Haupt S., Frazier A. (2021). Immunological memory to SARS-CoV-2 assessed for up to 8 months after infection. Science.

[19] McMahan K., Yu J., Mercado N.B., Loos C., Tostanoski L.H., Chandrashekar A., Liu J., Peter L., Atyeo C., Zhu A. (2021). Correlates of protection against SARS-CoV-2 in rhesus macaques. Nature.

[20] Quantiferon SARS-CoV-2. https://www.qiagen.com/gb/resources/resourcedetail?id=68fa6418-35fb-4e61-a0a2-37aa40dfce05&lang=en.

[21] Focosi D., Franchini M. (2021). COVID-19 neutralizing antibody-based therapies in humoral immune deficiencies: A narrative review. Transfus. Apher. Sci..

[22] Libster R., Perez Marc G., Wappner D., Coviello S., Bianchi A., Braem V., Esteban I., Caballero M.T., Wood C., Berrueta M. (2021). Early High-Titer Plasma Therapy to Prevent Severe Covid-19 in Older Adults. N. Engl. J. Med..

[23] Guthmiller J.J., Stovicek O., Wang J., Changrob S., Li L., Halfmann P., Zheng N.Y., Utset H., Stamper C.T., Dugan H.L. (2021). SARS-CoV-2 Infection Severity Is Linked to Superior Humoral Immunity against the Spike. mBio.

[24] Combes A.J., Courau T., Kuhn N.F., Hu K.H., Ray A., Chen W.S., Chew N.W., Cleary S.J., Kushnoor D., Reeder G.C. (2021). Global absence and targeting of protective immune states in severe COVID-19. Nature.

[25] Douglas A.P., Trubiano J.A., Barr I., Leung V., Slavin M.A., Tam C.S. (2017). Ibrutinib may impair serological responses to influenza vaccination. Haematologica.

[26] Solodky M.L., Galvez C., Russias B., Detourbet P., N’Guyen-Bonin V., Herr A.L., Zrounba P., Blay J.Y. (2020). Lower detection rates of SARS-COV2 antibodies in cancer patients versus health care workers after symptomatic COVID-19. Ann. Oncol..

[27] Herishanu Y., Avivi I., Aharon A., Shefer G., Levi S., Bronstein Y., Morales M., Ziv T., Shorer Arbel Y., Scarfò L. (2021). Efficacy of the BNT162b2 mRNA COVID-19 vaccine in patients with chronic lymphocytic leukemia. Blood.

[28] Parry H., McIlroy G., Bruton R., Ali M., Stephens C., Damery S., Otter A., McSkeane T., Rolfe H., Faustini S. (2021). Antibody responses after first and second Covid-19 vaccination in patients with chronic lymphocytic leukaemia. Blood Cancer J..

