# Supplementary material for: Convalescent Plasma in a Patient with Protracted COVID-19 and Secondary Hypogammaglobulinemia Due to Chronic Lymphocytic Leukemia: Buying Time to Develop Immunity?
Source: Infect Dis Rep. 2021 Sep 27;13(4):855–64. doi: 10.3390/idr13040077 (PMC8544405; doi:10.3390/idr13040077)
Supplement: Supplementary file 1 [file idr-13-00077-s001.zip › idr-1289327-supplementary.pdf]

SUPPLEMENTARY DATA WITH MANUSCRIPT

Convalescent plasma in a patient with protracted COVID-19 and secondary hypogammaglobulinemia due to chronic lymphocytic leukemia: buying time to develop cellular immunity?

Figure S1. Serology for BK polyomavirus and seasonal coronavirus 229E-N

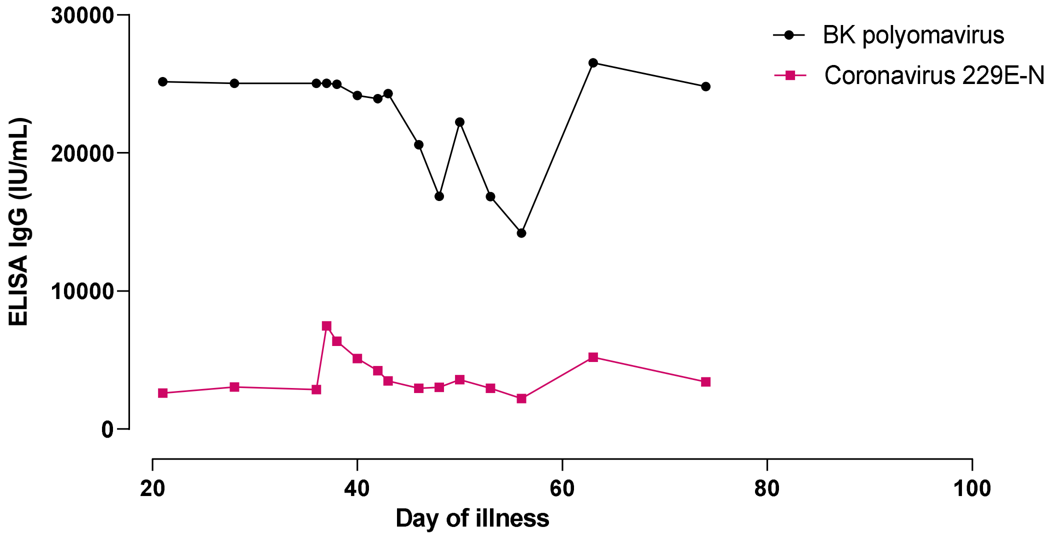

Figure S2. Anti-S antibodies (Wantai) in dilutions of plasma

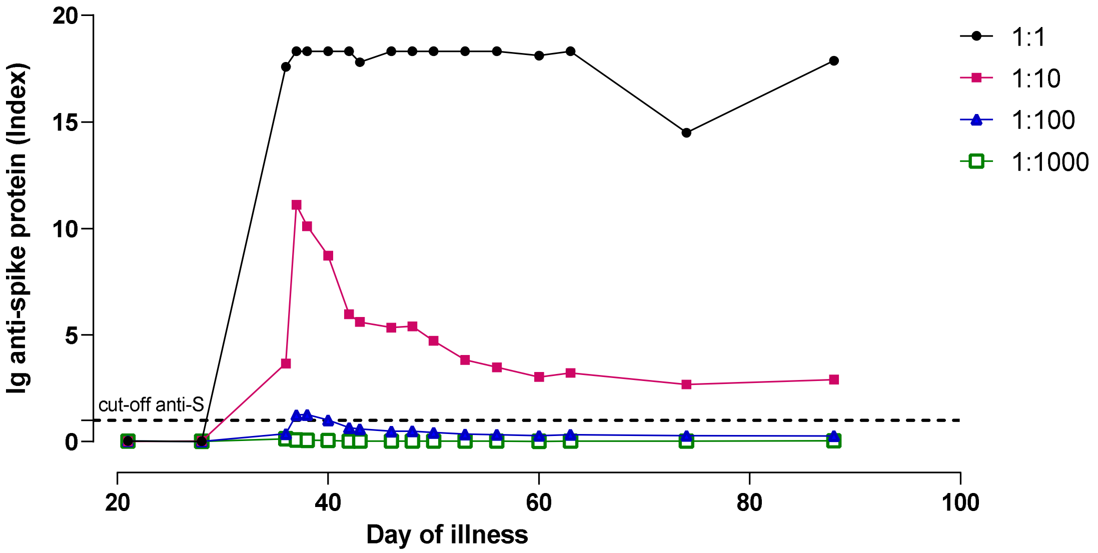

Methods T cell assays

Patient's blood was sampled on day 53 and 88 after disease onset and PBMCs were isolated using Ficoll-Isopaque. After cryopreservation, PBMCs were thawed and $1x10^6$ cells were cultured in the presence of in 100μL IMDM (Lonza) + 10% FCS (Sigma) + 1.4% L-glutamine (Lonza) + 1% Pen/Strep (Lonza) + 5 μg/mL Brefeldin A (Sigma). PBMCs were stimulated by supplementing culture conditions with 1μg/mL SARS-CoV-2 peptide pool derived from nucleocapsid (Miltenyi, cat#130-126-699), membrane (Miltenyi, cat#130-126-703), spike (Miltenyi, cat#130-126-701), 1% DMSO (negative control), or 1μg/mL CMV pp65 peptide pool (positive control). After overnight incubation, T cell frequency and activation was measured by staining for Zombie-Red (biolegend, cat#423110), CD4-Pe-Cy7 (Beckman Coulter, cat#737660), CD8-APC-H7 (BD Biosciences, cat#560179), Ki-67 (Dako, cat#F7268), HLA-DR (BD Biosciences, cat#560743), CD137-APC (BD Pharmingen, cat#550890), CD154-Pacific Blue (Biolegend, cat#310820), IFNγ-BV711 (BD Biosciences, cat#564039), TNFα-BV421 (BD Biosciences, cat#566275), PD1-BUV661 (BD Biosciences, cat#750260), and CD38-BV605 (BD Biosciences, cat#740401). Cells were stained after fixation using 1% paraformaldehyde and permeabilization using 0.1% saponin (Sigma) + 2mL 200 g/L albumin + 1% pen/strep (Lonza) in PBS. Samples were measured on a 5-laser Cytek Aurora and analyzed by FlowJo v10.7.1. and GraphPad 8.4.2.

Figure S3.

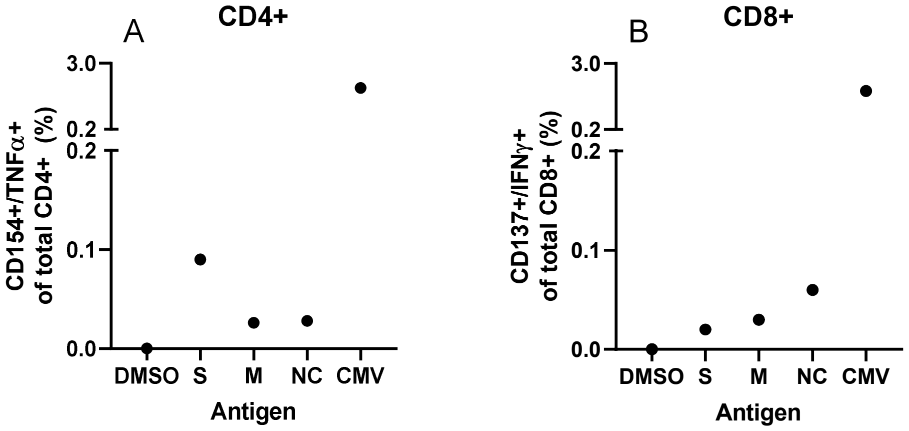

Reactivity of CD4+ and CD8+ T cells against SARS-CoV-2 proteins

FACS analysis of T cells obtained after overnight stimulation of PBMCs sampled at day 53 after symptom onset using peptides derived from SARS-CoV-2 spike (S), membrane (M), or nucleocapsid (NC) protein. Dimethylsulfoxide (DMSO) is used as a negative control, and cytomegalovirus (CMV) as positive control.

A. Frequency of CD4+ T cells specific for S, M, and NC is shown as % CD154+/TNFα+ of total CD4+ T cells.

B. Frequency of CD8+ T cells specific for S, M, and NC is shown as % CD137+/IFNγ+ of total CD8+ T cells.

Figure S4. Activation marker expression on CD4+ and CD8+ T cells.

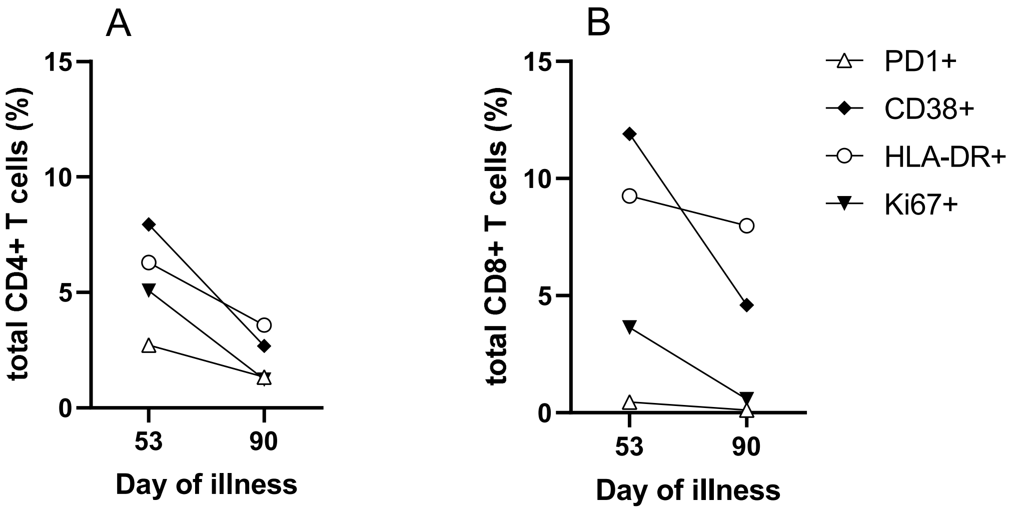

FACS analysis of T cells from PBMCs sampled on day 53 and 88 of illness.

A. Frequency of CD4+ T cells expressing activation markers PD1, CD38, HLA-DR or Ki67.

B. Frequency of CD8+ T cells expressing activation markers PD1, CD38, HLA-DR or Ki67.

Table S1. Whole genome sequencing of all nasopharyngeal samples with sufficient viral load (C_T <30)

Day of illness	Level	5'UTR	NSP1	NSP3						NSP4	NSP12		NSP13		NSP14	NSP16	Spike		NS3		NS7b	NS8	N*		
21	NT	C241T	C313T	C3037T	C3619T	G4510A	C5178T	5184C	C6541T	9133C	14077A	C14408T	C16646T	G17259T	G19480A	G21193T	C21846T	A23403G	23536C	C26060T	G27827T	G28086T	G28881A	G28882A	G28883C
	AA	-	-	-	-	-	T820I	-	-	-	-	P323L	T137M	E341D	G481S	D179Y	T95I	D614G	-	T223I	M24I	A65S	R203K		G204R
28	NT	C241T	C313T	C3037T	C3619T	G4510A	5178C	5184C	C6541T	9133C	14077A	C14408T	16646C	G17259T	G19480A	G21193T	C21846T	A23403G	23536C	C26060T	G27827T	G28086T	G28881A	G28882A	G28883C
	AA	-	-	-	-	-	-	-	-	-	-	P323L	-	E341D	G481S	D179Y	T95I	D614G	-	T223I	M24I	A65S	R203K		G204R
36	NT	C241T	C313T	C3037T	C3619T	G4510A	C5178T	5184C	C6541N	9133C	A14077G	C14408T	16646C	G17259T	G19480A	G21193T	C21846T	A23403G	23536C	C26060T	G27827N	G28086T	G28881A	G28882A	G28883C
	AA	-	-	-	-	-	T820I	-	-	-	N213D	P323L	-	E341D	G481S	D179Y	T95I	D614G	-	T223I	-	A65S	R203K		G204R
47	NT	C241T	C313T	C3037T	C3619T	G4510A	C5178T	5184C	C6541N	9133C	14077A	C14408T	16646C	G17259T	G19480A	G21193T	C21846T	A23403G	23536C	C26060T	G27827T	G28086T	G28881A	G28882A	G28883C
	AA	-	-	-	-	-	T820I	-	-	-	-	P323L	-	E341D	G481S	D179Y	T95I	D614G	-	T223I	M24I	A65S	R203K		G204R
53	NT	C241T	C313T	C3037T	C3619T	G4510A	5178C	C5184T	C6541T	9133C	14077A	C14408T	16646C	G17259T	G19480A	G21193T	C21846T	A23403G	C23536T	C26060T	G27827T	G28086T	G28881A	G28882A	G28883C
	AA	-	-	-	-	-	-	P822L	-	-	-	P323L	-	E341D	G481S	D179Y	T95I	D614G	-	T223I	M24I	A65S	R203K		G204R
63	NT	C241T	C313T	C3037T	C3619T	G4510A	5178C	C5184T	C6541T	C9133T	14077A	C14408T	16646C	G17259T	G19480A	G21193T	C21846T	A23403G	C23536T	C26060T	G27827T	G28086T	G28881A	G28882A	G28883C
	AA	-	-	-	-	-	-	P822L	-	-	-	P323L	-	E341D	G481S	D179Y	T95I	D614G	-	T223I	M24I	A65S	R203K		G204R

Abbreviations: AA indicates amino acid; NT indicates nucleotide

The initial SARS-CoV-2 genome sequence belonged to the B.1.1.209 lineage, with a total of 24 nucleotide substitutions throughout the genome compared to the original Wuhan-1 strain (10 synonymous mutations indicated in yellow shade and 14 non-synonymous mutations in blue).

– indicates no change in amino acid. The top line indicates the genomic regions of SARS-CoV-1. Cells marked in grey are regions that were not covered with sequencing, with N indicating that the nucleotide could not be determined (could be either A,T,C or G). During follow-up, non-synonymous substitutions detected in at least two consecutive isolates were observed at positions 5178 and 5184, resulting in T820I and P822L amino acid-replacements located in NSP3 involved in viral replication, but no non-synonymous mutations in the S protein were observed.

* In the N region, three nucleotide mutations led to two AA changes (both G28881A and G28882A are part of the triplet encoding AA R203K.
